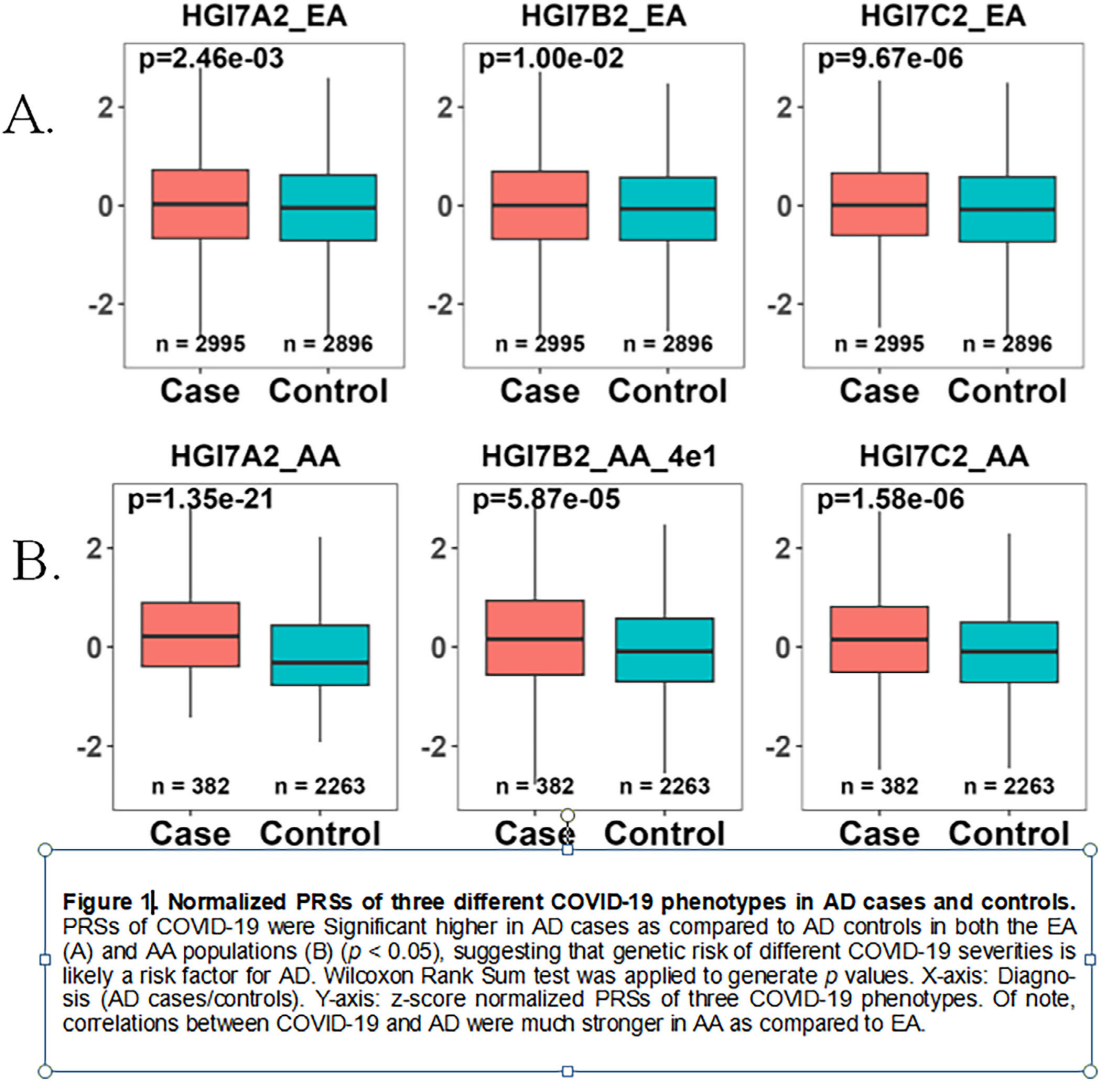# Shared Genetic Risk Factors Contribute to the High Comorbility between Alzheimer’s Disease and COVID‐19 in European and African Populations

**DOI:** 10.1002/alz.086755

**Published:** 2025-01-03

**Authors:** Jingchun Chen

**Affiliations:** ^1^ UNLV‐NIPM, Las Vegas, NV USA

## Abstract

**Background:**

The global outbreak of COVID‐19, caused by the SARS‐CoV‐2 virus, has been linked to long‐term neurological complications, including Alzheimer’s disease (AD) among seniors. However, the precise genetic impact of COVID‐19 on long‐term AD development remains unclear.

**Method:**

This study leveraged genome‐wide association study (GWAS) data and genotype data to explore the genetic correlation between AD and various COVID‐19 phenotypes across European ancestry (EA) and African ancestry (AA). We first calculated the polygenic risk scores (PRSs) of three COVID‐19 phenotypes in AD cases and controls from both EA and AA populations, then determined the genetic correlations between COVID‐19 PRSs and AD by logistic regression analyses with or without adjusting for age, sex, and *APOE* genotypes. Furthermore, shared SNPs and genes were identified through variant mapping and gene annotation from the large GWAS data of both diseases.

**Result:**

Our study identified significant positive correlations between AD diagnosis and COVID‐19 in both populations. Notably, correlations were stronger from AA as compared to the EA population. In addition, significant shared variants and genes were identified in the chr17q21.31 region, suggesting common biological mechanisms may contribute to the high comorbidity between AD and COVID‐19.

**Conclusion:**

Further functional studies are required to understand their causal relationships, leading to enhanced prevention and treatments for both conditions.